# MiR-24 Promotes the Survival of Hematopoietic Cells

**DOI:** 10.1371/journal.pone.0055406

**Published:** 2013-01-30

**Authors:** Tan Nguyen, Audrey Rich, Richard Dahl

**Affiliations:** 1 Department of Microbiology and Immunology, Indiana University School of Medicine, South Bend, Indiana, United States of America; 2 Cancer Research and Treatment Center, University of New Mexico Health Sciences Center, Albuquerque, New Mexico, United States of America; 3 Department of Biological Sciences, University of Notre Dame, Notre Dame, Indiana, United States of America; University of Sao Paulo – USP, Brazil

## Abstract

The microRNA, miR-24, inhibits B cell development and promotes myeloid development of hematopoietic progenitors. Differential regulation of cell survival in myeloid and lymphoid cells by miR-24 may explain how miR-24′s affects hematopoietic progenitors. MiR-24 is reported to regulate apoptosis, either positively or negatively depending on cell context. However, no role for miR-24 in regulating cell death has been previously described in blood cells. To examine miR-24′s effect on survival, we expressed miR-24 via retrovirus in hematopoietic cells and induced cell death with cytokine or serum withdrawal. We observed that miR-24 enhanced survival of myeloid and B cell lines as well as primary hematopoietic cells. Additionally, antagonizing miR-24 with shRNA in hematopoietic cells made them more sensitive to apoptotic stimuli, suggesting miR-24 functions normally to promote blood cell survival. Since we did not observe preferential protection of myeloid over B cells, miR-24′s pro-survival effect does not explain its promotion of myelopoiesis. Moreover, expression of pro-survival protein, Bcl-xL, did not mimic miR-24′s impact on cellular differentiation, further supporting this conclusion. Our results indicate that miR-24 is a critical regulator of hematopoietic cell survival. This observation has implications for leukemogenesis. Several miRNAs that regulate apoptosis have been shown to function as either tumor suppressors or oncogenes during leukemogenesis. MiR-24 is expressed highly in primary acute myelogenous leukemia, suggesting that its pro-survival activity could contribute to the transformation of hematopoietic cells.

## Introduction

Hematopoiesis is a life-long process critical for the development of cell types that are required for transporting oxygen and protecting from pathogens. All mature blood cells are derived from pluripotent hematopoietic stem cells (HSCs) that self-renew or differentiate into more committed, but still multipotent, progenitor cells[1]. These cells give rise to committed progenitors, which produce the mature functional cells of the hematopoietic system. Differentiation, proliferation, and survival of blood cells are tightly regulated within the bone marrow microenvironment[2]. Perturbations in these pathways can lead to the development of hematological malignancies[3].

MicroRNAs (miRNAs) have emerged as vital for proper hematopoiesis over the last decade[4], [5]. MiRNAs are a class of small (∼22 nucleotides) non-coding RNAs that regulate cell differentiation, proliferation, and survival pathways[4]. MiRNAs modulate gene expression through inhibiting the stability and translation of target mRNAs. Chen and colleagues first described the expression of miRNAs in the hematopoietic system, cloning approximately 100 miRNAs from mouse bone marrow[5]. We identified the *mrn23a* miRNA cluster (miRs-23a, 27a, and 24-2 expressed from single RNA transcript) as a transcriptional target of the essential hematopoietic factor, PU.1[6]. Expression of the entire cluster or just miR-24 mimics PU.1’s ability to promote myeloid (monocyte/granulocyte) differentiation of hematopoietic progenitor cells[6], [7].

MiR-24 is implicated in regulating apoptosis. Reported targets of miR-24 include pro-apoptotic proteins (FAF-1, Caspase 9, Bim and Apaf-1)[8], [9], [10], [11], [12], [13], [14] with miR-24 expression associated with survival. Conversely, miR-24 has also been shown to target pro-survival genes such as PAK4 and Bcl-2, which could lead to increased cell death[15], [16]. There is clearly a discrepancy as to whether miR-24 promotes cell survival or cell death. Which role it favors may depend on cell specific environments. A role for miR-24 in regulating survival of hematopoietic cells has not been previously reported. Since miR-24 may have distinct effects on survival depending on cell context, we hypothesized that differential effects on apoptotic regulation in lymphoid versus myeloid cells could explain the myeloid expansion we observed when miR-24 is exogenously expressed in hematopoietic progenitors[6].

In this study we investigated whether miR-24 affects hematopoietic cell survival. Results from hematopoietic cell lines and primary mouse hematopoietic cells demonstrated that miR-24 enhances cell survival. Exogenous expression of miR-24 decreased protein levels of Caspase 9 and Bim, whereas knockdown of miR-24 resulted in increased expression of these pro-apoptotic factors. Furthermore this activity was observed in both myelocytes and lymphocytes, suggesting that regulation of cell death does not significantly contribute to miR-24's preferential promotion of myelopoiesis over lymphopoiesis[6]. Additionally, expression of the pro-survival gene Bcl-xL in hematopoietic cultures did not mimic miR-24. Several miRNAs that regulate cell death act as tumor suppressors or oncogenes during leukemogenesis[17]. A potential role for miR-24 as a leukemic oncogene is discussed.

## Materials and Methods

### Ethics statement

For experiments performed with primary hematopoietic cells, mouse bone marrow was used as the source of the cells. The use of mice in these experiments was approved by the Indiana University School of Medicine and University of Notre Dame IACUCs (Protocol # 13-017).

### Cell Culture

70Z/3 and MPRO cell lines were obtained from ATCC (Manassas, VA). The 293FT line was obtained from Invitrogen (Carlsbad, CA). 32Dcl3 was a gift from Allan Friedman (Johns Hopkins)[18], [19]. Unless otherwise stated, the following cell culture media and additives were obtained from Invitrogen (Carlsbad, CA). 70Z/3 cells were grown in RPMI supplemented with 10% FBS, 0.1 mM glutamax, 10 mM HEPES, and 1 mM sodium pyruvate. 32Dcl3 cells were grown in IMDM supplemented with 10% FBS, 10% Wehi-3B conditioned media, 55 µM 2-mercaptoethanol (BME). MPRO cells were cultured in IMDM, 20% horse serum, 10% HM5 conditioned media, and 55 µM BME. 293FT cells were grown in Opti-MEM, and 5% FBS. OP9s were cultured in alpha MEM, 20% FBS, sodium pyruvate, and 55 µM BME. All medias contained 50 U/ml penicillin, and 50 µg/ml streptomycin

Bone marrow cells were isolated from tibias and femurs of 6-week old mice. Mature erythroid cells were removed by ammonium chloride lysis. Nucleated cells were lineage depleted with a MACS lineage cell separation kit according to manufacturer's instructions (Miltenyi Biotec, Auburn, CA). Bone marrow was infected with retrovirus through 2 rounds of spinoculation. During infection, cells were cultured in IMDM supplemented with 10% defined FBS, 55 µM BME, 50 U/ml penicillin, streptomycin, 0.1 mM Glutamax, 10 ng/mL mIL-3, 10 ng/mL mIL-6, 50 ng/mL mSCF, and 7 µg/mL polybrene. Recombinant mouse cytokines were obtained from R&D Systems or Invitrogen. For apoptotic evaluation, GFP+ cells were sorted 4d post spinoculation and after 4 additional days, apoptosis was induced by cytokine and serum withdrawal for 24h. For evaluating B cell versus myeloid development, infected cells were co-cultured with OP9 cells for 12 days in IMDM media containing 1 ng/mL mIL-7 and 5 ng/ml mFlt3L as previously described[6].

### Cell Transduction/Transfection

MSCV-GFP and MSCV-GFP miRNA expressing retroviral plasmids were co-transfected into 293FT cells together with the retroviral packaging vector pCL-Eco (Imgenex, San Diego, CA) using Lipofectamine 2000 (Invitrogen, Carlsbad, CA). ShRNA antagonist of miR-24 (miArrest miRNA inhibitor) was expressed in a lentiviral vector co-expressing mCherry and puromycin resistance (GeneCopoeia, Rockville, MD). pVSV-G, pMDL, and pRSV-REV plasmids (National Gene Vector Biorepository, Indianapolis, IN) were co-transfected into 293FT cells with Fugene HD Transfection Reagent (Promega Corporation, Madison, WI). 48 h and 72 h post-transfection viral supernatants were harvested and concentrated using Centricon Plus-70 filters (Millipore, Billerica, MA). Hematopoietic cells were transduced with either retroviral or lentiviral particles in their respective media, containing 7 µg/mL polybrene (Millipore, Billerica, MA). Stable cell lines were generated by sorting for GFP+ cells or selecting for puromycin resistance.

### Immunoblots

Whole-cell extracts were prepared by lysing cells in RIPA buffer (50 mM Tris pH 7.5, 150 mM NaCl, 1% NP-40, 0.5% EDTA, 0.1% SDS and protease inhibitor cocktail (Roche)). Protein concentration was determined using the Bicinchoninic Acid (BCA) protein assay (Pierce, Rockford, IL). 50 μg of whole cell lysates was separated by SDS-PAGE and transferred to nitrocellulose membrane. Membranes were blotted with the following antibodies: Bim, Caspase 9, GAPDH, and β-actin (Cell Signaling, Danvers, MA). Horseradish peroxidase conjugated secondary antibodies were purchased from GE Healthcare UK Ltd (Buckinghamshire, England). SuperSignal West Femto Substrate enabled detection of antibodies (Thermo Scientific, Rockford, IL). Experiments were done in triplicate and densitometry analysis was performed using BIORAD Chemidoc XRS+ System using Imager Lab Software (Hercules, CA) or ImageJ (NIH, Bethesda, MD).

### Quantitative Real-time Polymerase Chain Reaction (QRT-PCR)

Total RNA was prepared using TRIzol (Invitrogen, Carlsbad, CA) according to the manufacturer's protocol. Complementary DNA (cDNA) was reverse transcribed from 10 ng of RNA using Taqman microRNA reverse transcription kit according to manufacturer's protocol (Applied Biosystems, Carlsbad, CA). For QRT-PCR, we used miR-specific Taqman primers obtained from Applied Biosystems. Expression level of miR-24 was normalized to 18S rRNA expression (Millipore, Billerica, MA). All experiments were performed in triplicate using BioRad CFX96 C1000 System (BioRad, Hercules, CA). ΔΔC_T_ calculations were used to normalize signal versus 18S rRNA as the control.

### Flow Cytometry

In the apoptosis and differentiation experiments, cells were washed twice with cold phosphate buffered saline (PBS; 137 mM M NaCl, 2.7 mM KCl, and 11.9 mM phosphate buffer, pH 7.4) and stained with Annexin V-PE and 7-AAD (BD Biosciences, San Diego, CA) or CD19-PE (Invitrogen, Carlsbad, CA) and CD11b-APC (eBioscience, San Diego, CA) following manufacturer's procedure. Stained cells were subsequently assessed using Beckman Coulter FC500 Flow Cytometer (Brea, CA) and data was analyzed using Flowjo software (Tree Star, Ashland, OR).

### Statistical Analysis

Data were analyzed using a paired 2-tailed Student's t-test. *P* value < 0.05 was considered significant.

## Results

### Expression of miR-24 antagonizes apoptosis in hematopoietic cells

To determine if miR-24 regulates cell death in hematopoietic cells, we generated MPRO (myeloid) and 70Z/3 (pre-B) cell lines that stably express miR-24. Cells were infected with control MSCV-GFP or MSCV-miR24 retroviruses. Infected cells were isolated by cell sorting for GFP expression. Taqman analysis was used to confirm expression of miR-24 (**Fig S1A**)[6]. Apoptosis was induced by recombinant GM-CSF withdrawal from the MPRO cell line and by serum withdrawal from the 70Z/3 cell line.

**Figure 1:**
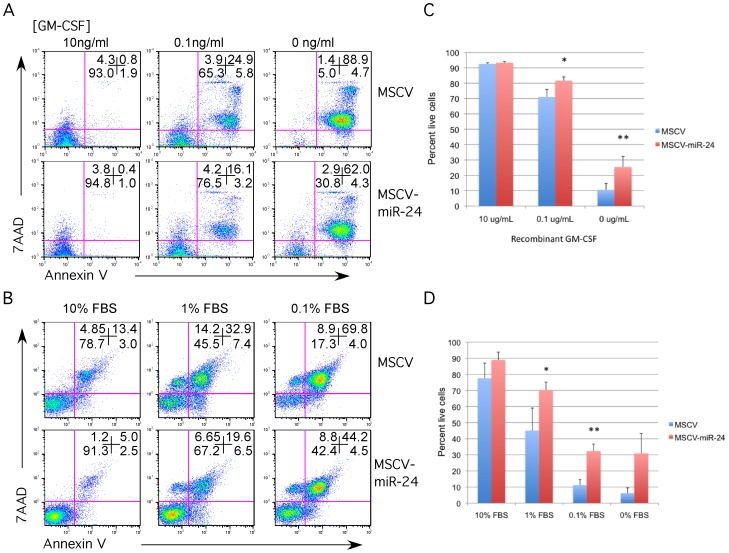
MiR-24 inhibits apoptosis in both myeloid and lymphoid cell lines. GM-CSF dependent MPRO myeloid cells and 70Z/3 pre B cells were infected with MSCV-GFP or MSCV-miR-24 retrovirus. Infected cells were isolated by fluorescent cell sorting for GFP. For both MPRO and 70Z/3 cells, apoptosis was examined by flow cytometry using fluorescently labeled annexin V and the cell permeability dye 7AAD. **A**) MPRO cells were washed out of 10 ng/ml GM-CSF media and replated in media containing the indicated amounts of GM-CSF for 48 h in order to induce apoptosis. **B**) Percent live cells determined for each condition is shown. Error bars represent standard error of the mean (SEM). For each condition N = 5. Differences between MSCV and miR-24 infected cells were determined to be significant at 0.1 and 0 ng/ml GM-CSF by paired student t-test with p values of *<0.025, and **<0.015. **C**) 70Z/3 cells were switched to media containing 1%, 0.1%, or 0% FBS and cultured for 48 h to induce apoptosis. **D**) Percent live cells determined for each condition is shown. Error bars represent SEM. For each condition N = 4. Differences were determined to be significant at 1% and 0.1% FCS by paired student t-test with p values of *<0.03, and **<0.01.

MPRO cells were cultured in 10 ng/ml (maintenance concentration), 0.1 ng/ml and 0 ng/ml GM-CSF for 48 h. Apoptosis was evaluated by flow cytometry using Annexin V-PE staining and 7AAD exclusion. Expression of miR-24 was associated with a significant increase in cell survival (Annexin V+, 7AAD+) at both 0.1 ng/ml and 0 ng/mL GM-CSF compared to MSCV empty vector control cells (**Fig 1A,B**). Similarly, when we induced cell death in the 70Z/3 pre-B cell line by withdrawing serum for 48 h, we observed that the miR-24 expressing cells survived significantly better than control-infected cells (**Fig 1C,D**). In addition, we observed that miR-24 inhibited cell death from cytokine withdrawal in the SCF (Stem Cell Factor) dependent hematopoietic stem cell line EML (**Fig S2**). These results indicate that miR-24 protects both myeloid and B cell lines from apoptosis. We did not observe a consistent effect on cell survival when we expressed miR-27a in MPRO, or 70Z/3 cells demonstrating that miR-24's pro-survival activity is not due to overexpressing any miscellaneous miRNA (**Fig S3**).

**Figure 2:**
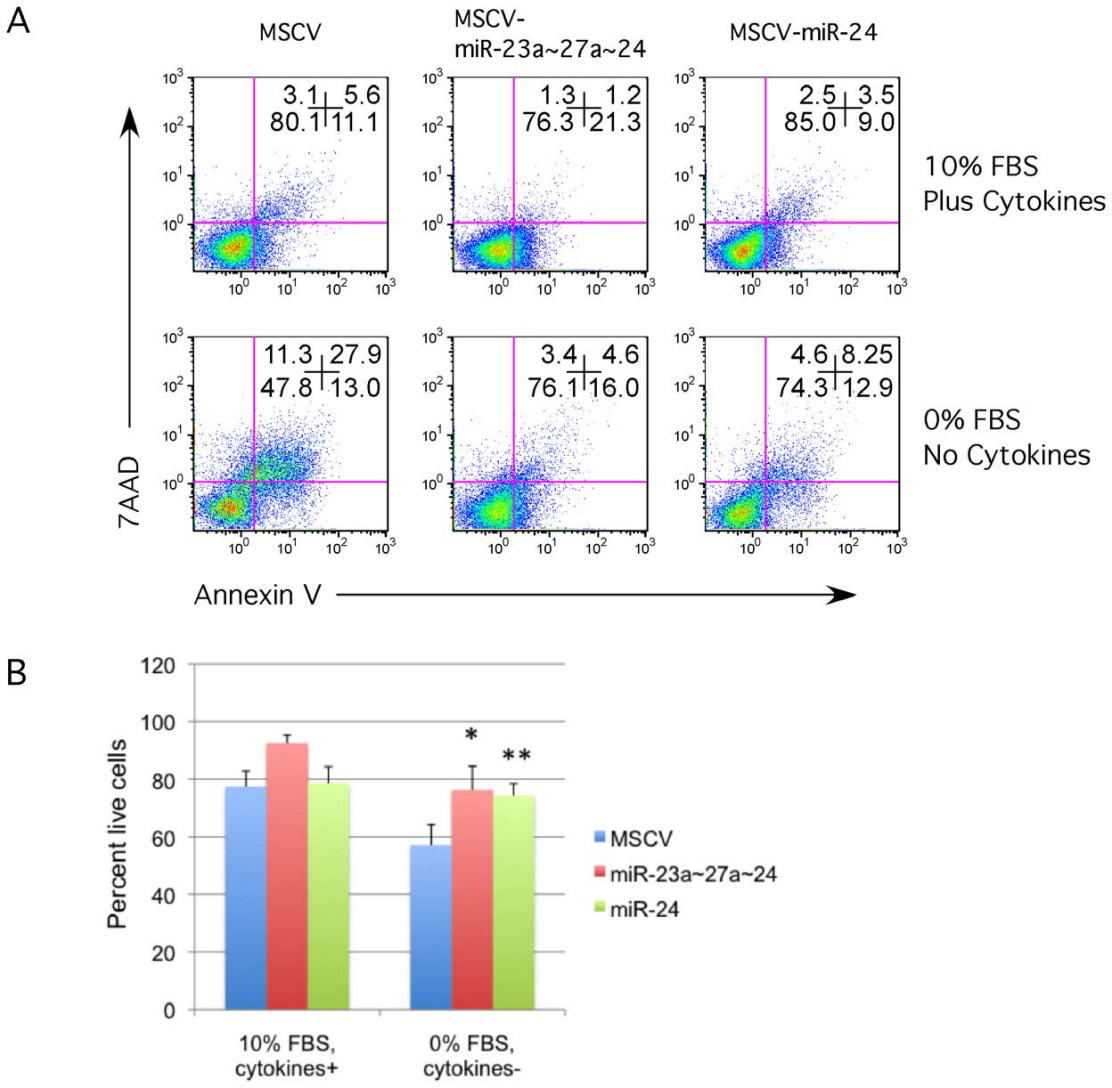
MiR-24 inhibits apoptosis in primary mouse hematopoietic progenitors. Lineage negative hematopoietic progenitors were isolated from 6-week-old C57/BL6j mice by magnetic separation (Miltenyi Biotec). Progenitors were infected with the indicated retrovirus for 48 h in media containing SCF, IL6, and IL3. GFP+ cells were isolated by FACs. Cells were cultured for 6d post-infection in media containing the cytokines listed previously. Cells were withdrawn from serum and cytokines for 24 h to induce cell death. **A**) Apoptosis was examined by flow cytometry using fluorescently labeled annexin V and the cell permeability dye 7AAD. **B**) Percent live cells determined for each condition is shown. Error bars represent standard error of the mean (SEM). For each condition N = 3. Differences were determined to be significant for both MSCV-miR-23a∼27a∼24, and miR-24 infected cells by paired student t-test with p values of *<0.02, and **<0.02.

The ability of miR-24 to protect primary hematopoietic cells from apoptotic stimuli was subsequently investigated. Lineage negative mouse bone marrow cells were infected with miR-24 and empty vector control viruses similar to the cell lines above. Cells were cultured in media containing serum and the cytokines SCF, IL-6, and IL-3. After 4d, GFP+ cells were isolated by cell sorting. Taqman analysis indicated that cells infected with miR-24 virus expressed approximately two-fold higher levels of miR-24 compared to control virus-infected cells **(Fig S1B**). GFP+ cells were cultured an additional 96 h and then withdrawn from serum and cytokines for 24 h. We observed nearly a 60% decrease in cell death in miR-24 and miR-23a cluster (miRs-23a∼27a∼24) expressing cells compared to the MSCV infected cells (**Fig 2**).

### MiR-24 targets pro-apoptotic proteins Bim and Caspase 9 in hematopoietic cells

**Figure 3:**
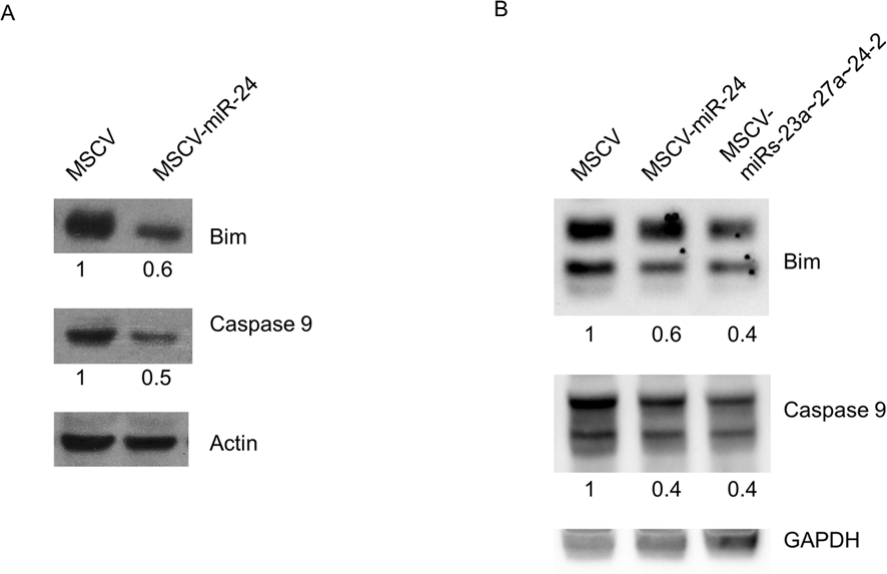
MiR-24 decreases the levels of pro-apoptotic proteins Bim and Caspase 9. Whole cell lysates were prepared from **A**) MPRO cells infected with MCSV-GFP or MSCV-miR-24, or **B**) 70Z/3 cells infected with MSCV-GFP, MSCV-miR-24 or MSCV-miRs-23a∼27a∼24. Lysates were separated by SDS-PAGE and immunoblotted. Blots were probed with antibodies to Bim, Caspase 9, actin and/or GAPDH. Protein expression was normalized to either actin or GAPDH expression. Fold protein expression compared to MSCV-GFP infected cells is shown.

In primary mouse cardiomyocytes and Xenopus retina, it was demonstrated that miR-24 inhibited apoptosis through targeting the pro-apoptotic proteins Bim and Caspase 9[12], [14]. We performed western blots to examine whether these proteins are targeted in hematopoietic cells as well. We found that pro-apoptotic proteins Bim and Caspase 9 were both reduced by approximately 2-fold when miR-24 was overexpressed in MPRO and 70Z/3 cells (**Fig 3**). Additionally we see decreases in Bim and Caspase 9 when we co-express miR-24 with its cluster partners miR-23a, and miR-27a, which would be the way the endogenous miR-24 gene would be expressed. This suggests that miR-24 is at least partially promoting cell survival by down-regulating these pro-apoptotic proteins.

### Knockdown of miR-24 makes cells more susceptible to apoptotic stimuli

**Figure 4:**
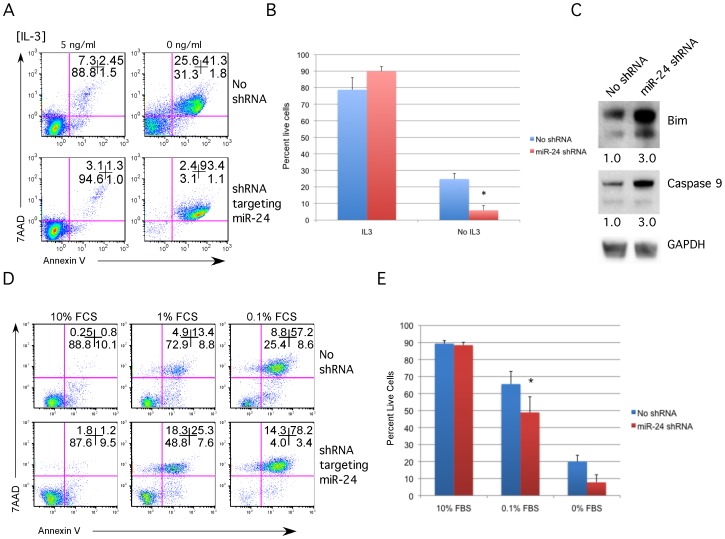
Decreased miR-24 sensitizes cells to apoptotic stimuli, and increases pro-apoptotic protein expression. IL-3 dependent myeloid 32Dcl3 cells and 70Z/3 pre-B cells were infected with lentivirus expressing an shRNA inactivating miR-24, and puromycin resistance. Infected cells were selected in puromycin and decreased expression of miR-24 validated by quantitative RT-PCR (**Fig S4**). **A**) 32Dcl3 and knockdown cells were washed out of 10 ng/ml IL-3 containing media. Cells were replated for 48 h in media containing 10 ng/ml or 0 ng/ml IL-3. Apoptosis was examined by flow cytometry using fluorescently labeled annexin V and the cell permeability dye 7AAD. **B**) Percent live cells determined for each condition is shown. Error bars represent standard error of the mean (SEM). For each condition N = 5. Differences were determined to be significant under no IL-3 condition by unpaired student t-test with a p value of *<0.01. **C**) Whole cell lysates were prepared from 32cl3 and miR-24 knockdown cells. Lysates were separated by SDS-PAGE and immunoblotted. Blots were probed with antibodies to Bim, Caspase 9, and GAPDH. Protein expression was normalized to GAPDH expression. Fold protein expression compared to the cells not expressing miR-24 shRNA is shown. **D**) 70Z/3 and knockdown cells were washed out of 10 ng/ml IL-3 containing media. Cells were replated for 48 h in media containing 10% FCS or 0% FCS. Apoptosis was examined by flow cytometry using fluorescently labeled annexin V and the cell permeability dye 7AAD. **E**) Percent live cells determined for each condition is shown. Error bars represent SEM. For each condition N = 3. Differences were determined to be significant at 0.1% FCS by unpaired student t-test with a p value of *<0.05.

Overexpression of miR-24 may promote pro-survival effects that the endogenous miR-24 does not perform. To examine this, we repeated the apoptosis assay and protein analysis in cell lines we generated to underexpress miR-24. Knockdown cells were generated by stably infecting cells with a lentivirus that expresses an shRNA antagonizing miR-24. We generated miR-24 knockdown in 70Z/3 cells and the IL3-dependent myeloid 32Dcl3 cell line. We were unable to generate stable knockdown of miR-24 in the MPRO myeloid cells. 32Dcl3 cells like MPRO cells are a mouse myeloid progenitor cell line, which can be induced to differentiate into mature granulocytes[20]. Taqman analysis demonstrated over 2-fold reductions in endogenous miR-24 levels in the 70Z/3 and 32Dcl3 cells expressing the shRNA (**Fig S4**). This may under represent the decrease in active miR-24 as we may still be detecting inactive miR-24 bound to the expressed shRNA. Antagonizing endogenous miR-24 had the opposite effect of overexpression. 32Dcl3 cells expressing miR-24 shRNA demonstrated increased cell death when withdrawn from IL-3 containing media (**Fig 4A,B**). Similarly we observed a significant decrease in cell survival of 70Z/3 cells cultured in 0.1% FCS (**Fig 4D,E**). Additionally we observed that decreasing miR-24 activity in 32Dcl3 cells resulted in a 3-fold increase in Bim and Capase-9 protein levels (**Fig 4C**).

### Inhibition of apoptosis in hematopoietic progenitors does not promote myelopoiesis

**Figure 5:**
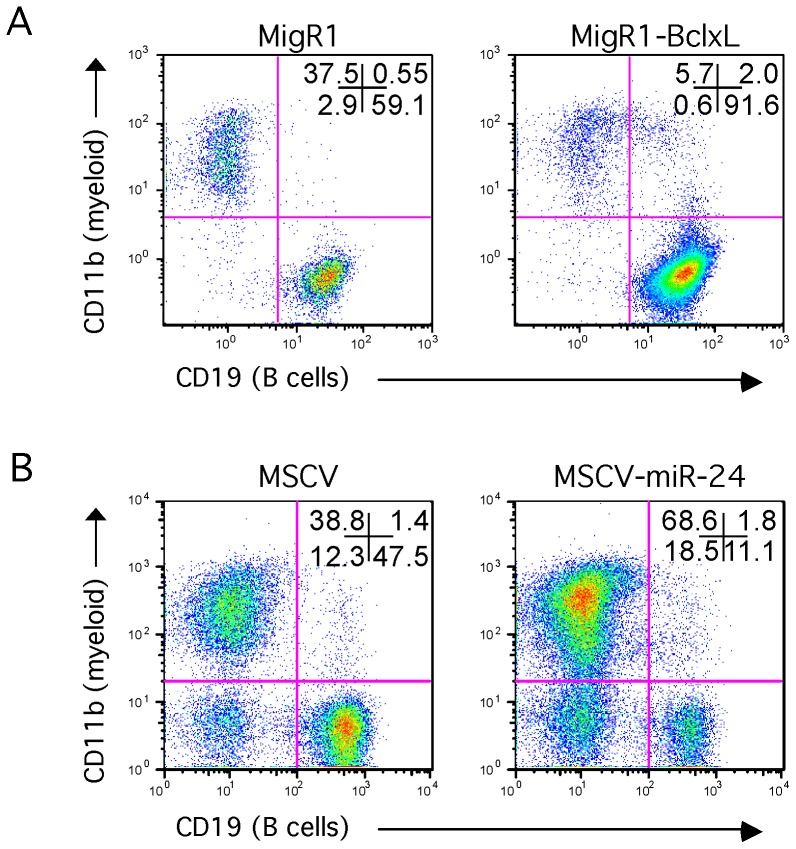
Inhibition of apoptosis does not skew hematopoietic progenitor development on OP9 stromal cells to myelopoiesis. Lin- mouse hematopoietic progenitors were infected with the indicated MSCV-based retroviruses expressing (co-expressing) GFP. Cells were cultured on OP9 cells 12d with IL-7 and Flt3L. Differentiation was analyzed by flow cytometry using fluorescently labeled antibodies that recognize the cell surface proteins CD19 (B cells), and CD11b (myeloid; macrophages and granulocytes). **A**) Bone marrow cells were infected with MigR1 or MigR1-Bcl-xL retroviruses. N = 2. *P<05. **B**) For comparison bone marrow cells were infected with MSCV or MSCV-miR-24 retrovirus. As previously observed miR-24 expression in bone marrow cells increased the production of CD11b+ myeloid cells, and decreased the percentage of CD19+ B cells[6].

MiR-24 promotes myelopoiesis and inhibits lymphopoiesis when expressed in mouse hematopoietic progenitors[6]. Potentially miR-24′s pro-survival effect could contribute to this phenotype. To determine if a pro-survival activity could affect the balance of lymphoid and myeloid cells in our culture system, we infected hematopoietic progenitors with a Bcl-xL expressing retrovirus. Bcl-xL is an anti-apoptotic protein that protects both myeloid and lymphoid cells from apoptosis[21], [22]. If protection from apoptosis contributes to the increased myelopoiesis we previously observed, we expect that Bcl-xL could mimic this effect. Bcl-xL and control virus infected progenitors were cultured on OP9 stromal cells as we previously did with miR-24 -infected cells[6]. After 12d of culture, lymphoid and myeloid differentiation was evaluated by CD19 and CD11b cell surface expression. We did not observe an increase in CD11b+ myeloid cells or a decrease in CD19+ lymphoid cells as we did with miR-24 expression (**Fig 5**). It appeared that Bcl-xL had an effect opposite of miR-24, enhancing lymphoid development in the OP9 cultures.

## Discussion

Previous studies have linked miR-24 to regulating apoptosis, both positively and negatively. Expression of miR-24 in liver, gastric, prostate, and cervical cancer cell lines protects these cells from apoptosis whereas knockdown of miR-24 sensitizes them to apoptosis[13], [23]. During development miR-24 protects cells from apoptosis in the Xenopus retina and murine cardiomyocytes[12], [14]. In other cellular contexts miR-24 was observed to be pro-apoptotic. In MCF-7 breast cancer cells miR-24 targets pro-survival protein Bcl-2 leading to increased sensitivity to apoptotic stimuli[16], [24]. MiR-24 induces the death of primary endothelial cells through repressing expression of the PAK4 kinase which phosphorylates and inactivates the pro-apoptotic protein BAD[15]. These reports demonstrate the importance of cell context on miR-24 function.

We previously published that miR-24 promotes myelopoiesis and inhibits B lymphopoiesis during adult blood development[6]. Since miR-24 may either positively or negatively affect cell survival depending on cell context, we were interested in determining if miR-24 promotes survival of myeloid cells and/or induced apoptosis of lymphoid cells. We observed that miR-24 expression in blood cells partially protects from cell death induced by cytokine and/or serum withdrawal. Consistent with this observation, miR-24 expression decreased the protein levels of pro-apoptotic proteins Caspase 9 and Bim, which were previous identified as miR-24 targets in non-hematopoietic systems[12], [14]. In blood we observed that knockdown of endogenous miR-24 increases the protein levels of Bim and Caspase 9, suggesting that miR-24 normally functions to regulate the levels of these proteins.

Although we observed that miR-24 expression enhances the survival of hematopoietic cells, we did not observe differential effects on myeloid and lymphoid cells, which could have explained the effects of miR-24 on myelo- and lymphopoiesis. Additionally expression of the pro-survival protein Bcl-xL in hematopoietic progenitors did not mimic the effect of miR-24 expression. We conclude that modulation of apoptosis is not responsible for changes in myeloid and lymphoid composition of our hematopoietic cultures[6]. MiR-24 may promote myelopoiesis and/or inhibit lymphopoiesis through affecting cell cycle regulation or cell fate adoption of stem and progenitor cells. Known targets of MiR-24 include several cell cycle inhibitors (p27Kip1, p57Kip2, and p16INK4a)[8], [9], [10], [11], [12], [13]. Additionally, it has been shown that exogenous miR-24 enhances the proliferation of hematopoietic cell lines K562 and 32D[25]. However, our preliminary results from BrdU incorporation assays has not revealed differences in proliferation of primary mouse myeloid or lymphoid cells infected with miR-24 retrovirus (Data not shown). The cell line study evaluated total cell number so some of the increase in cells they observed may be due to miR-24 decreasing cell death. There are several examples of miRNAs determining hematopoietic cell fate decisions of stem and progenitor cells through regulation of transcription factor expression. MiR-17-92 miRNA cluster inhibits myelopoiesis through antagonizing RUNX1, and miR-150 promotes megakaryopoiesis through antagonizing c-myb[26], [27]. In 32D cells exogenous miR-24 was shown to block myeloid differentiation[25]. Paradoxically in this study endogenous miR-24 along with the clustered miRNA miR-23a and miR-27a increased expression during 32D differentiation into granulocytes. We are currently investigating whether miR-24 directs the cell fate acquisition of hematopoietic progenitors through modulating gene expression. Intriguingly, miR-24 expressed in a pre-B cell line results in the upregulation of several myeloid specific genes including *CSF1R*, *Lyz*, *FcgRIII*, *CCL9*, and *SFPI1* (PU.1), and downregulation of B cells genes *Pax5*, *EBF1*, and *IgHγ* (Unpublished data).

MiR-24′s regulation of cell death implicates it as a gene involved in leukemia initiation and/or maintenance. Several miRNAs contribute to tumorigenesis through targeting apoptotic mechanisms[17]. MiRs -15a and -16-1 are well characterized as chronic lymphocytic leukemia (CLL) tumor suppressors through the targeting of Bcl-2[28]. Both genes are deleted or repressed in the majority of primary CLL samples examined. MiR-32 targeting of Bim protects acute myelogenous leukemia (AML) cells from apoptosis by chemotherapeutics[29]. MiR-24 is highly expressed in primary human AML samples, suggesting that antagonizing miR-24 in AML cells could enhance their susceptibility to apoptotic stimuli including current chemotherapeutics[30].

In conclusion, we have shown for the first time that miR-24 promotes hematopoietic cell survival. This appears to be an important normal function of miR-24, as knockdown of miR-24 sensitized cells to apoptotic stimuli. Others have shown that miR-24 expression is increased by growth factor signaling so miR-24 may assist in the pro-survival affects of growth factors and cytokines by keeping levels of pro-apoptotic proteins like Caspase 9 and Bim low[31]. Analogous to other pro-survival miRNAs, misregulation of miR-24 may contribute to the survival of malignant hematopoietic cells. However miR-24′s regulation of apoptosis does not appear to significantly contribute to miR-24′s promotion of myelopoiesis and inhibition of lymphopoiesis.

## Supporting Information

Figure S1 **Expression of miR-24 in virally transduced 70Z/3 cells.** MiR-24 gene expression analysis is shown from cells infected with retroviruses encoding the indicated miRNAs. GFP+ cells were isolated and RNA extracted. MiR-Taqman quantitative RT-PCR assays were conducted to quantify miR-24 expression. Expression was normalized to 18S rRNA. A) Gene expression is shown from 70Z/3 cells that were infected with the indicated retroviruses. Expression levels are relative to miR-24 levels in 70Z/3 cells infected with the control MSCV retrovirus. Expression of miR-24 in MSCV and MSCV-miR-24 infected MPRO cells was published previously[6]. Infection of MPRO cells with the miR-24 containing virus results in an almost 3-fold increase in miR-24 compared to control-infected MPRO cells. Data represented as mean ±SEM. N = 3. *P<05. B) Lineage depleted mouse bone marrow cells were infected with the indicated retroviruses. GFP+ cells were isolated by FACs. MiR-24 expression is relative to bone marrow cells infected with control retrovirus. Data represented as mean ±SEM. N = 3. *P<05.
(TIF)

Figure S2 **MiR-24 inhibits apoptosis in the SCF dependent EML hematopoietic stem cell line.** SCF dependent EML cells were infected with MSCV-GFP or MSCV-miR-24 retrovirus. Infected cells were isolated by fluorescent cell sorting for GFP. EML cells were washed out of SCF media and replated in media containing the indicated amounts of SCF for 48 h in order to induce apoptosis. Cell death was examined by flow cytometry using fluorescently labeled annexin V and the cell permeability dye 7AAD.
(TIF)

Figure S3 **MiR-27a does not increase cell survival in hematopoietic cell lines.** GM-CSF dependent MPRO myeloid cells and 70Z/3 pre B cells were infected with MSCV-GFP or MSCV-miR-27a retrovirus. Infected cells were isolated by fluorescent cell sorting for GFP. **A**) MPRO cells were washed out of 10 ng/ml GM-CSF media and replated in media containing the indicated amounts of GM-CSF for 48 h in order to induce apoptosis. **B**) 70Z/3 cells were switched to media containing 1%, 0.1%, or 0% FBS and cultured for 48 h to induce apoptosis. For both MPRO and 70Z/3 cells, apoptosis was examined by flow cytometry using fluorescently labeled annexin V and the cell permeability dye 7AAD.
(TIF)

Figure S4 **MiR-24 knockdown in myeloid and B cells.** 32Dcl3 myeloid cells and 70Z/3 pre-B cells were infected with a puromycin resistant lentivirus that expresses an shRNA that targets miR-24. Stably infected cells were selected in puromycin. RNA was isolated and miR-24 Taqman assays performed. RNA expression was normalized to Sno202 expression. A. Fold expression compared to 32Dcl3 cells not expressing the miR-24 shRNA is shown. Data represented as mean ±SEM. N = 3. ***P<0005. B. Fold expression compared to 70Z/3 cells not expressing the miR-24 shRNA is shown. Data represented as mean ±SEM. N = 3. *P<015.
(TIF)
