# Supplementary figures and images for: MiR-24 Promotes the Survival of Hematopoietic Cells
Source: PLoS One. 2013 Jan 30;8(1):e55406. doi: 10.1371/journal.pone.0055406 (PMC3559586; doi:10.1371/journal.pone.0055406)

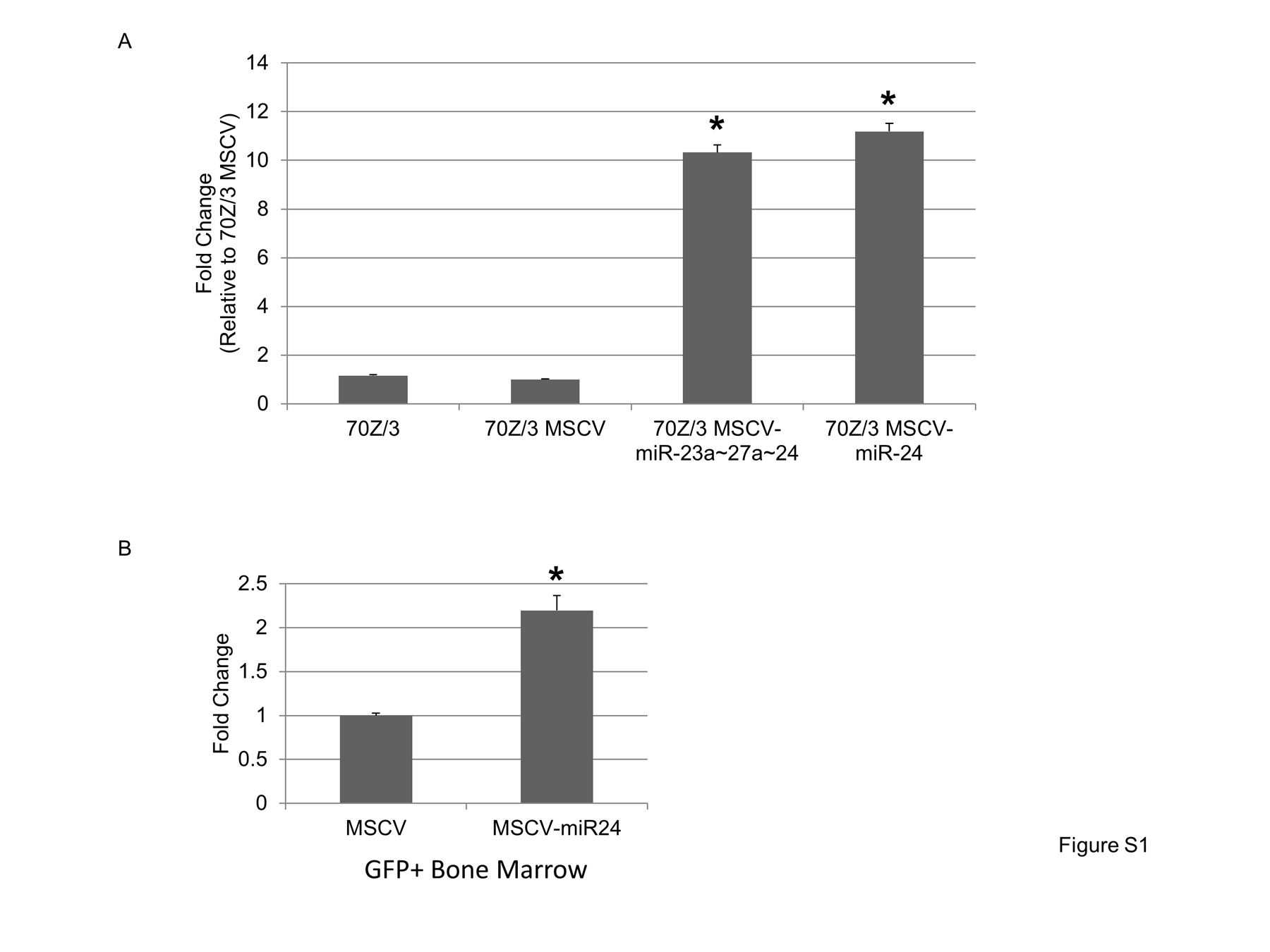

Supplement: Figure S1 — Expression of miR-24 in virally transduced 70Z/3 cells. MiR-24 gene expression analysis is shown from cells infected with retroviruses encoding the indicated miRNAs. GFP+ cells were isolated and RNA extracted. MiR-Taqman quantitative RT-PCR assays were conducted to quantify miR-24 expression. Expression was normalized to 18S rRNA. A) Gene expression is shown from 70Z/3 cells that were infected with the indicated retroviruses. Expression levels are relative to miR-24 levels in 70Z/3 cells infected with the control MSCV retrovirus. Expression of miR-24 in MSCV and MSCV-miR-24 infected MPRO cells was published previously[6]. Infection of MPRO cells with the miR-24 containing virus results in an almost 3-fold increase in miR-24 compared to control-infected MPRO cells. Data represented as mean ±SEM. N = 3. *P<05. B) Lineage depleted mouse bone marrow cells were infected with the indicated retroviruses. GFP+ cells were isolated by FACs. MiR-24 expression is relative to bone marrow cells infected with control retrovirus. Data represented as mean ±SEM. N = 3. *P<05. (TIF) [file pone.0055406.s001.tif]

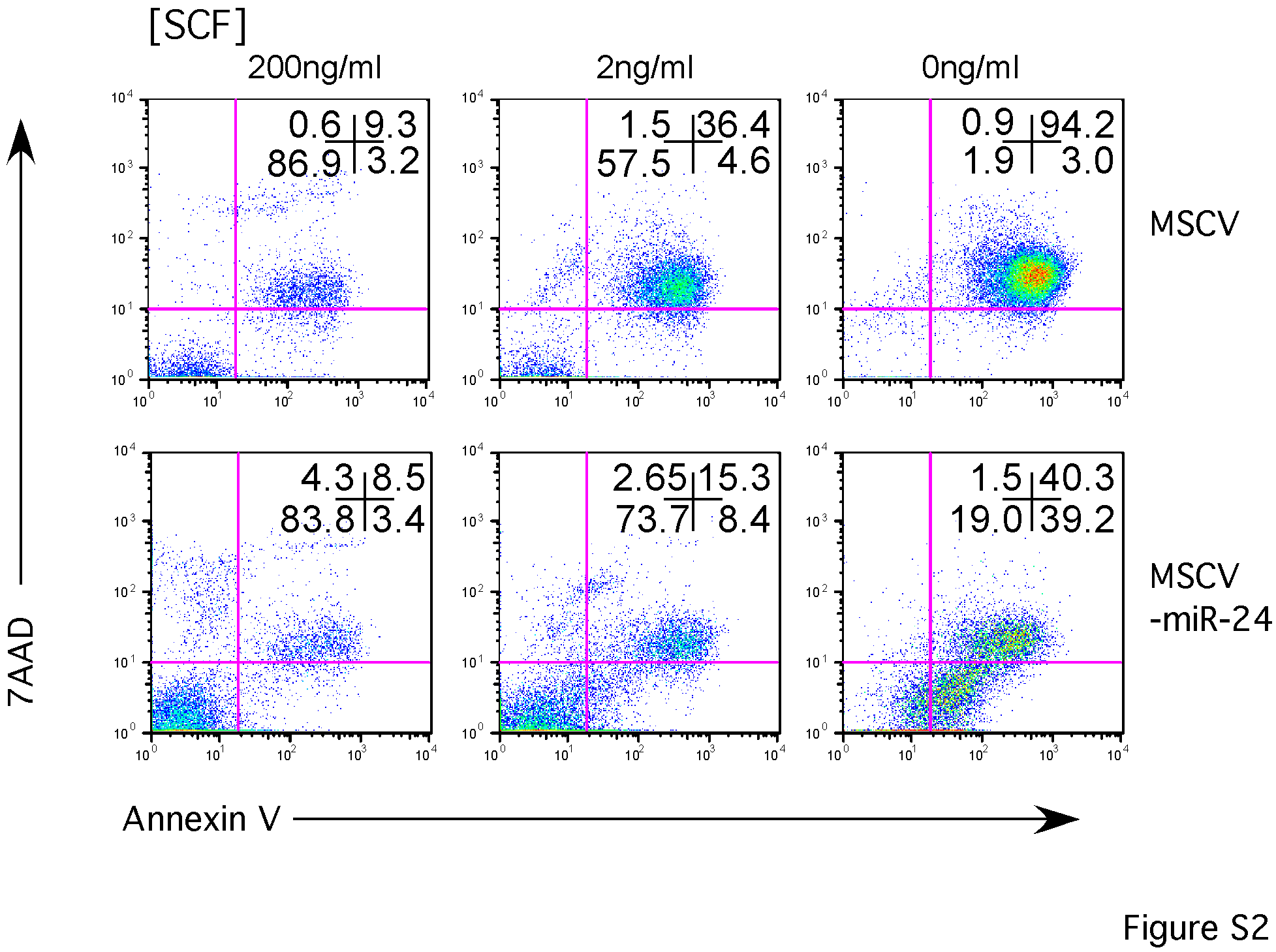

Supplement: Figure S2 — MiR-24 inhibits apoptosis in the SCF dependent EML hematopoietic stem cell line. SCF dependent EML cells were infected with MSCV-GFP or MSCV-miR-24 retrovirus. Infected cells were isolated by fluorescent cell sorting for GFP. EML cells were washed out of SCF media and replated in media containing the indicated amounts of SCF for 48 h in order to induce apoptosis. Cell death was examined by flow cytometry using fluorescently labeled annexin V and the cell permeability dye 7AAD. (TIF) [file pone.0055406.s002.tif]

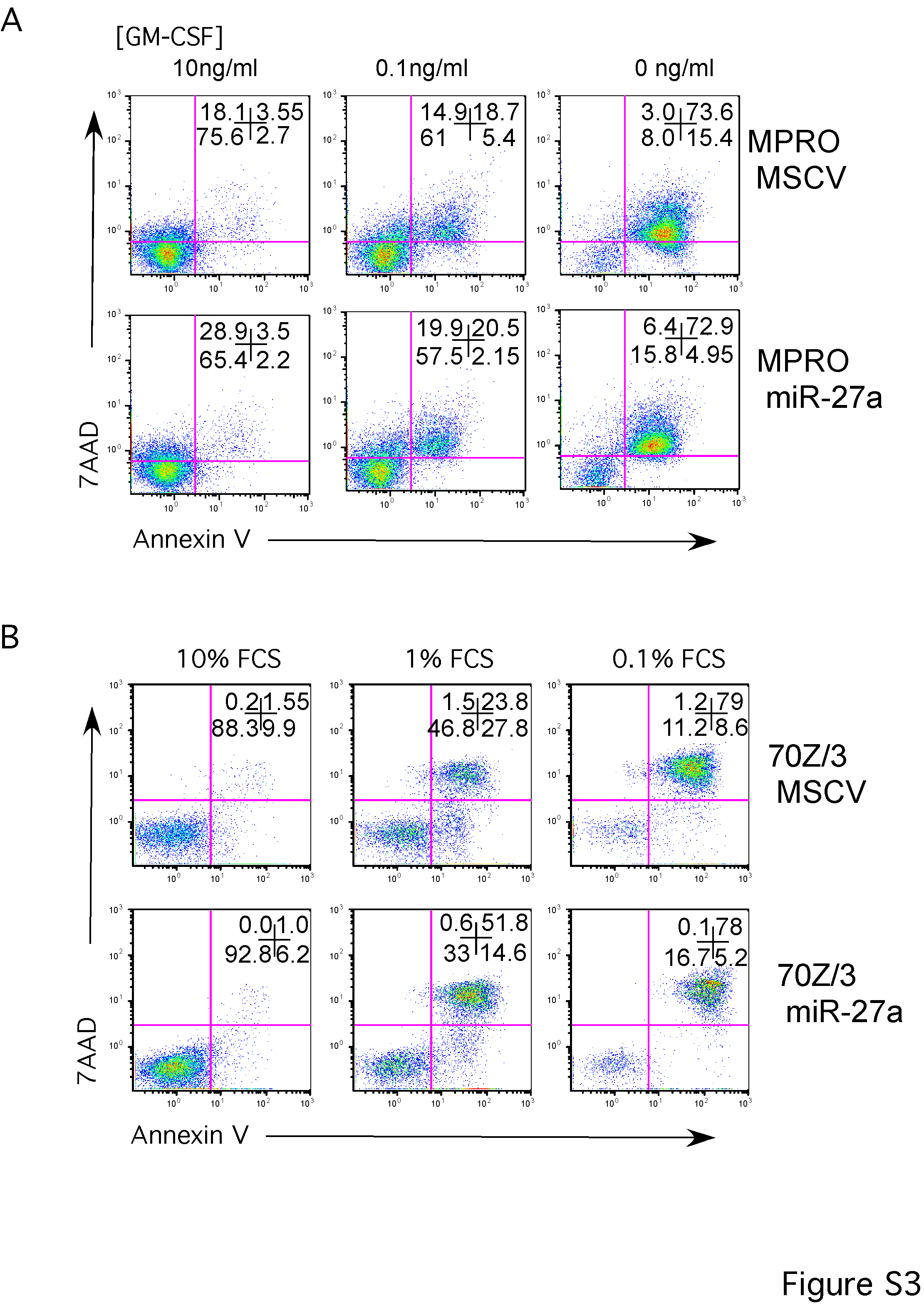

Supplement: Figure S3 — MiR-27a does not increase cell survival in hematopoietic cell lines. GM-CSF dependent MPRO myeloid cells and 70Z/3 pre B cells were infected with MSCV-GFP or MSCV-miR-27a retrovirus. Infected cells were isolated by fluorescent cell sorting for GFP. A) MPRO cells were washed out of 10 ng/ml GM-CSF media and replated in media containing the indicated amounts of GM-CSF for 48 h in order to induce apoptosis. B) 70Z/3 cells were switched to media containing 1%, 0.1%, or 0% FBS and cultured for 48 h to induce apoptosis. For both MPRO and 70Z/3 cells, apoptosis was examined by flow cytometry using fluorescently labeled annexin V and the cell permeability dye 7AAD. (TIF) [file pone.0055406.s003.tif]

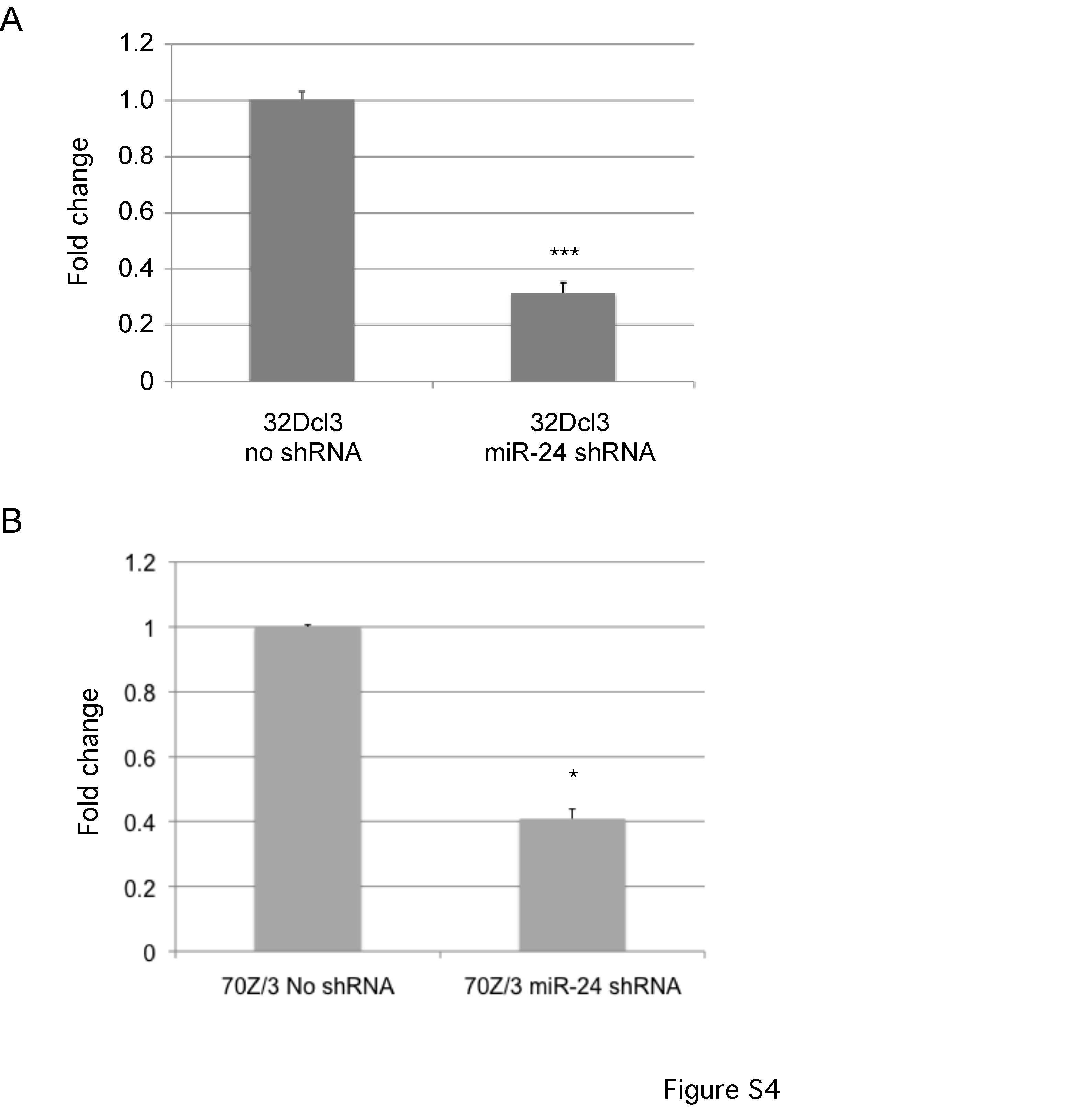

Supplement: Figure S4 — MiR-24 knockdown in myeloid and B cells. 32Dcl3 myeloid cells and 70Z/3 pre-B cells were infected with a puromycin resistant lentivirus that expresses an shRNA that targets miR-24. Stably infected cells were selected in puromycin. RNA was isolated and miR-24 Taqman assays performed. RNA expression was normalized to Sno202 expression. A. Fold expression compared to 32Dcl3 cells not expressing the miR-24 shRNA is shown. Data represented as mean ±SEM. N = 3. ***P<0005. B. Fold expression compared to 70Z/3 cells not expressing the miR-24 shRNA is shown. Data represented as mean ±SEM. N = 3. *P<015. (TIF) [file pone.0055406.s004.tif]
